# Progressive changes in non-coding RNA profile in leucocytes with age

**DOI:** 10.18632/aging.101220

**Published:** 2017-04-27

**Authors:** Maider Muñoz-Culla, Haritz Irizar, Ana Gorostidi, Ainhoa Alberro, Iñaki Osorio-Querejeta, Javier Ruiz-Martínez, Javier Olascoaga, Adolfo López de Munain, David Otaegui

**Affiliations:** ^1^ Multiple Sclerosis Group, Biodonostia Health Research institute, San Sebastian, Spain; ^2^ Neuroscience Area, Biodonostia Health Research institute, San Sebastian, Spain; ^3^ REEM, Red Española de Esclerosis Múltiple, Spanish Network on Multiple Sclerosis, 08028 Barcelona, Spain; ^4^ Institute for Genomics and Multiscale Biology, Department of Genetics and Genomics Science, Icahn School of Medicine at Mount Sinai, New York, NY 10029, USA; ^5^ Parkinson Group, Biodonostia Health Research Institute, San Sebastian, Spain; ^6^ Genomic Platform, Biodonostia Health Research Institute, San Sebastian, Spain; ^7^ Neurology Department, Universitary Hospital Donostia, San Sebastian, Spain; ^8^ University of the Basque Country (UPV-EHU), Department of Neuroscience, San Sebastian, Spain; ^9^ IBERNED, Center for Biomedical Research in Network on Neurodegenerative Diseases, 28049 Madrid, Spain

**Keywords:** microRNA, non-coding RNA, transcriptome, regulation, aging, human, leucocytes

## Abstract

It has been observed that immune cell deterioration occurs in the elderly, as well as a chronic low-grade inflammation called inflammaging. These cellular changes must be driven by numerous changes in gene expression and in fact, both protein-coding and non-coding RNA expression alterations have been observed in peripheral blood mononuclear cells from elder people. In the present work we have studied the expression of small non-coding RNA (microRNA and small nucleolar RNA -snoRNA-) from healthy individuals from 24 to 79 years old. We have observed that the expression of 69 non-coding RNAs (56 microRNAs and 13 snoRNAs) changes progressively with chronological age. According to our results, the age range from 47 to 54 is critical given that it is the period when the expression trend (increasing or decreasing) of age-related small non-coding RNAs is more pronounced. Furthermore, age-related miRNAs regulate genes that are involved in immune, cell cycle and cancer-related processes, which had already been associated to human aging. Therefore, human aging could be studied as a result of progressive molecular changes, and different age ranges should be analysed to cover the whole aging process.

## INTRODUCTION

In the last years, aging has become one of the most studied biological processes, due to its increasing incidence in our population and also to its correlation to other highly prevalent diseases, such as cancer or neurodegenerative diseases [1]. It has been estimated that 30 % of the total population in Europe will be older than 65 years in 2050, becoming a huge challenge from the medical and social point of view.

From a biological viewpoint, aging has been defined as a progressive postmaturational decline in physiological capacity, accompanied by an increased susceptibility to disease and an increased mortality risk [2].

It is a complex and multifactorial process in which genetic, lifestyle and environmental factors play a role. The genetic configuration of each individual defines how the organism faces our lifestyle and the interaction with the environment in the aging process [1]. Aging could be defined as the phenotypic expression of cellular senescence in our tissues. Cellular senescence is characterized by cell cycle arrest and it is associated to major changes in gene expression patterns. Moreover, aging reaches most tissues and organs, which is measured as accumulation of senescent cells [3].

A high amount of cells of the immune system acquire senescent features, in the process termed immunosenescence [4]. These age-related changes result in increased susceptibility to viral and bacterial infection, poor vaccination response and higher incidence of agerelated diseases, as stated before. Apart from immune cell deterioration, a chronic low-grade inflammation has also been described in the elderly, the so called inflammaging. Inflammaging results from immune dysfunction and accumulation of senescent cells that promote proinflammatory signals, such as elevated secretion of proinflammatory cytokines and activation of NK-κB transcription factor [5,6].

These cellular and serological changes must be driven by numerous changes in gene expression. Previous works have shown that genomic DNA methylation declines with age [7] and also that there is a change in gene expression at transcriptomic level [8] in peripheral blood cells. Additionally, cellular changes could be led by products of the non-coding part of the genome, non-coding RNAs (ncRNAs).

ncRNAs include a wide variety of RNAs that can be classified according to their size in long ncRNAs (>200 bp) or small ncRNAs (<200 bp). Apart from the well-known ribosomal RNA or transfer RNA, other less characterized ncRNAs are being described to be involved in many cellular processes through the regulation of gene expression. This regulation can be achieved taking part in different processes, such as transcription, mRNA processing or epigenetic control [9].

Among small ncRNA family (sncRNA), microRNAs (miRNA) are the best described class of ncRNAs. They also participate in gene expression regulation, through the binding of target mRNA, triggering either their destabilization or translation inhibition. During the last decade, the implication of miRNAs in virtually all biological processes has been reported, including immune cell development, function [10–12] and immunosenescence [13].

Moreover, several works have studied miRNA expression in peripheral blood cells from donors of different ages, including preterm infants, young, adults, octogenarians and centenarians, in order to find healthy aging miRNA expression patterns [14–18]. Three of these reports, include centenarians in their studies, adding a valuable information about human healthy aging [15–17]. Conversely, Noren Hooten and colleagues compare old subjects (mean age = 64.5 years) with young subjects (mean age = 30 years) [14] while Lai et al compare preterm infants with adults (mean age= 37 years old)[18]. In all these works, the strategy to find age-related miRNAs has been to compare the expression pattern from the oldest subjects to that from youngest subjects.

Small non-coding RNAs include other subtypes apart from miRNAs and, among them, the small nucleolar RNAs (snoRNAs) have been the most widely studied. They perform sequence-specific 2′ -O-methylation and pseudouridylation of ribosomal RNA which takes place in the nucleolus after forming the small nucleolar ribonucleoprotein (snoRNPs) complex [19]. A role for snoRNAs in cancer has been described and they have also been proposed as good candidates for involvement in other diseases [20]. Recently, it has been reported the presence of snoRNA in human plasma samples and identified a subset of 9 snoRNA showing a decreased expression in individuals being older than 66.5 years [21]. This work supports the idea that several types of non-coding RNA may play a role in human aging.

In the present work, we wanted to focus on how blood miRNA expression changes progressively across the life course, from adulthood to the elderly, apart from the classical analysis of comparing elder subjects' expression profile to that of young subjects'. Using a miRNA microarray, we analyzed the expression of 1769 human miRNA and other sncRNAs and we identified 69 sncRNAs that progressively changed with age. Furthermore, this analysis suggests that 50 years could be a turning point in human aging process.

## RESULTS

**Figure 1:**
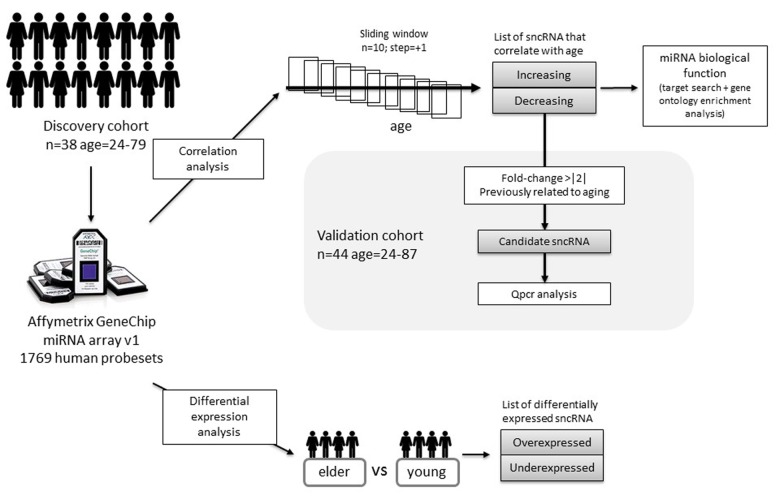
Study design and analysis workflow followed in the present study.

**Figure 2:**
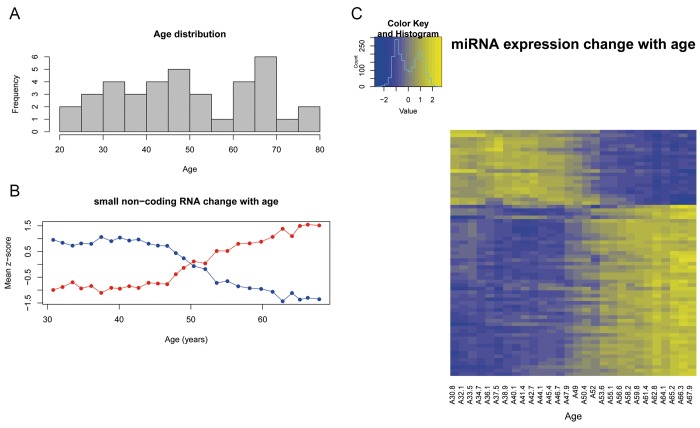
(**A**) Age distribution of subjects included in the study. (**B**) Expression of the sncRNA that progresively change with age according to the correlation analysis. y-axis represents the mean z-score values of the sncRNAs that correlate with age. Blue line represents the decreasing sncRNAs and red line represents the increasing sncRNAs. (**C**) Heatmap that represents the z-score value of the expression of the 69 sncRNAs correlated with age. Columns represent the mean age of the 28 sliding windows and rows are the 69 sncRNAs.

In the present work we wanted to explore how the global expression of sncRNA changes in human aging. To do that, we analysed the expression of sncRNA by microarrays in leucocytes from healthy donors of different ages, ranging from 24 years to 79 years old (Figure 2A). We took two approaches to analyse data. On the one hand, we performed a correlation analysis aiming at identifying progressively changing sncRNA and on the other hand, we applied a more classical differential expression analysis. Finally, we carried out validation experiments both in the same discovery cohort and in an independent cohort of 44 donors (age range: 24-87). This study design, which is depicted in Figure 1, allowed us to study the expression of sncRNA in all the stages of the adulthood and part of the elderly.

### Correlation and differential expression analysis

**Figure 3:**
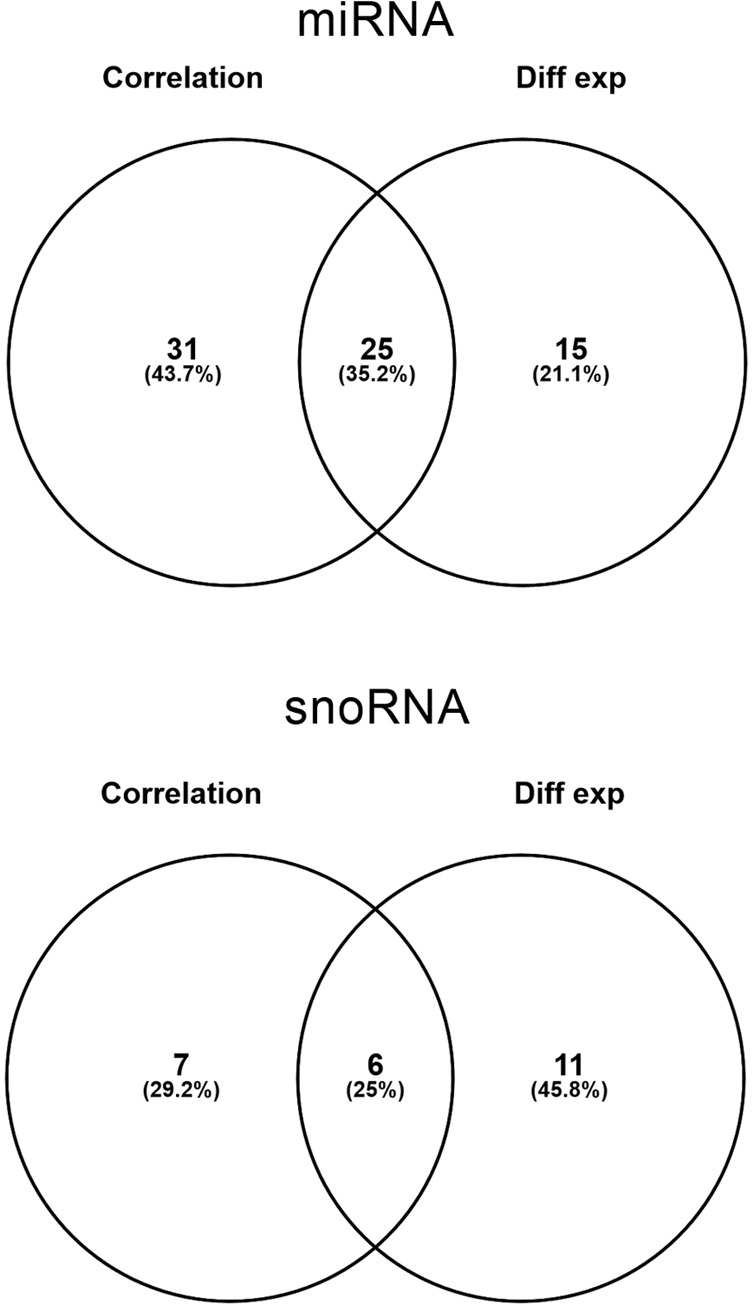
Number of miRNA (top) and snoRNA (bottom) identified by correlation and differential expression analysis and the overlapping probes between two analyses.

The first approach was to assess how the global sncRNA expression changes with chronological age and whether the sncRNA expression was correlated with human aging. The correlation analysis between gene expression and age resulted in 69 sncRNAs (56 miRNA and 13 snoRNA) (Table 1) (Figure 3) that progressively change with age (R>0.75) and have a fold-change greater than 1.5 between the oldest and the youngest sample. Forty-eight sncRNAs show an increasing expression pattern (R>0.75) while 21 have a decreasing expression pattern (R<-0.75) (Figure 2C). The representation of the mean z-score of expression for the decreasing and increasing sncRNAs (Figure 2B), shows that at 50 years old the two lines cross, highlighting that this could be a critical age.

**Table 1:** List of age-related small non-coding RNAs identified by correlation analysis ordered by Pearson correlation coefficient (Pearson R). Fold-change between the last and first sliding window and the expression trend are also shown.

Probeset Name	Type	Pearson R	FC (last/first)	Trend with age
U38B_st	CDBox	−0.947	0.364	decreasing
hsa-miR-125b_st	miRNA	−0.941	0.569	decreasing
hsa-miR-146a_st	miRNA	−0.917	0.561	decreasing
hsa-miR-19b_st	miRNA	−0.913	0.530	decreasing
hsa-miR-194_st	miRNA	−0.906	0.656	decreasing
hsa-miR-29a_st	miRNA	−0.902	0.655	decreasing
hsa-miR-130a_st	miRNA	−0.901	0.628	decreasing
hsa-let-7g_st	miRNA	−0.899	0.434	decreasing
hsa-let-7a_st	miRNA	−0.897	0.578	decreasing
hsa-miR-146b-5p_st	miRNA	−0.896	0.457	decreasing
hsa-miR-25_st	miRNA	−0.890	0.574	decreasing
U38B_x_st	CDBox	−0.884	0.457	decreasing
hsa-miR-15a_st	miRNA	−0.874	0.465	decreasing
hsa-miR-126_st	miRNA	−0.871	0.554	decreasing
hsa-miR-363_st	miRNA	−0.865	0.607	decreasing
hsa-let-7i_st	miRNA	−0.853	0.498	decreasing
hsa-miR-629_st	miRNA	−0.847	0.612	decreasing
hsa-miR-584_st	miRNA	−0.841	0.592	decreasing
hsa-miR-30b_st	miRNA	−0.839	0.476	decreasing
ACA40_x_st	HAcaBox	−0.800	0.282	decreasing
ACA8_x_st	HAcaBox	−0.781	0.644	decreasing
hsa-miR-1271_st	miRNA	0.768	1.497	increasing
U48_st	CDBox	0.771	1.601	increasing
ENSG00000212523_x_st	snoRNA	0.776	1.670	increasing
hsa-miR-500-star_st	miRNA	0.811	1.616	increasing
U46_st	CDBox	0.820	1.510	increasing
U25_st	CDBox	0.826	1.642	increasing
U43_x_st	CDBox	0.831	1.539	increasing
U43_st	CDBox	0.838	1.557	increasing
U91_s_st	scaRna	0.840	1.884	increasing
hsa-miR-1280_st	miRNA	0.848	1.659	increasing
ACA16_st	HAcaBox	0.853	1.498	increasing
hsa-miR-339-5p_st	miRNA	0.856	1.561	increasing
HBII-180A_x_st	CDBox	0.858	1.616	increasing
hsa-miR-663_st	miRNA	0.865	1.500	increasing
hsa-miR-1308_st	miRNA	0.869	1.604	increasing
hsa-miR-25-star_st	miRNA	0.878	1.658	increasing
hsa-miR-1275_st	miRNA	0.883	1.855	increasing
hsa-miR-149-star_st	miRNA	0.884	1.503	increasing
hsa-miR-1228-star_st	miRNA	0.888	1.685	increasing
hsa-miR-766_st	miRNA	0.894	1.730	increasing
hsa-miR-197_st	miRNA	0.894	1.657	increasing
hsa-miR-345_st	miRNA	0.897	1.683	increasing
hsa-miR-574-5p_st	miRNA	0.898	1.585	increasing
hsa-miR-505-star_st	miRNA	0.907	1.535	increasing
hsa-miR-423-3p_st	miRNA	0.909	1.656	increasing
hsa-miR-150-star_st	miRNA	0.913	1.716	increasing
hsa-miR-432_st	miRNA	0.921	2.327	increasing
hsa-miR-1281_st	miRNA	0.921	2.778	increasing
hsa-miR-877_st	miRNA	0.923	1.889	increasing
hsa-miR-486-3p_st	miRNA	0.925	1.678	increasing
hsa-miR-491-5p_st	miRNA	0.928	1.929	increasing
hsa-miR-23a-star_st	miRNA	0.929	2.524	increasing
hsa-miR-339-3p_st	miRNA	0.929	1.520	increasing
hsa-miR-502-3p_st	miRNA	0.931	1.759	increasing
hsa-miR-1307_st	miRNA	0.938	1.836	increasing
hsa-miR-328_st	miRNA	0.944	1.883	increasing
hsa-miR-500_st	miRNA	0.946	2.460	increasing
hsa-miR-744_st	miRNA	0.949	1.845	increasing
hsa-miR-193a-5p_st	miRNA	0.952	2.166	increasing
hsa-miR-574-3p_st	miRNA	0.954	1.811	increasing
hsa-miR-330-3p_st	miRNA	0.954	1.953	increasing
hsa-miR-941_st	miRNA	0.954	2.395	increasing
hsa-miR-425-star_st	miRNA	0.963	1.653	increasing
hsa-miR-423-5p_st	miRNA	0.963	1.998	increasing
hsa-miR-1301_st	miRNA	0.969	1.813	increasing
hsa-miR-378_st	miRNA	0.975	1.537	increasing
hsa-miR-501-5p_st	miRNA	0.978	2.688	increasing
hsa-miR-93-star_st	miRNA	0.982	1.646	increasing

In a second approach, we applied a classical differential expression analysis, comparing the expression of sncRNA between elder and young donors. To set up the age at which we should consider the segregation of two groups, we looked at the correlation analysis. According to those results, 50 years seems to be critical, so we set this age as the cut-off between the two groups. Therefore, we had 21 young samples (age ≤ 50) and 17 elder samples (age >50).

Differential expression analysis identified 39 overexpressed and 18 underexpressed sncRNAs in the elder group compared to the young group (57 sncRNAs in total) (limma test, FDR corrected p<0.05)(Table 2). Among those, there are 40 miRNA and 17 snoRNA.

**Table 2:** List of differentially expressed small non-coding RNAs between elder donors and young donors resulted from Limma test. sncRNAs are ordered by increasing fold-change.

Probeset	Type	adj.P.Val	FC (elder/young)	Change with age
hsa-miR-199a-3p_st	miRNA	0.016454	0.331	DOWN
hsa-miR-628-3p_st	miRNA	0.015405	0.385	DOWN
hsa-miR-30e-star_st	miRNA	0.032863	0.393	DOWN
hsa-let-7i_st	miRNA	0.004641	0.455	DOWN
hsa-miR-146b-5p_st	miRNA	0.040513	0.466	DOWN
hsa-let-7g_st	miRNA	0.015405	0.468	DOWN
hsa-miR-584_st	miRNA	0.014044	0.524	DOWN
hsa-miR-25_st	miRNA	0.006466	0.547	DOWN
hsa-let-7a_st	miRNA	0.001976	0.554	DOWN
hsa-miR-126_st	miRNA	0.032863	0.555	DOWN
hsa-miR-629_st	miRNA	0.015405	0.562	DOWN
hsa-miR-98_st	miRNA	0.032863	0.582	DOWN
hsa-miR-26b-star_st	miRNA	0.040513	0.583	DOWN
hsa-miR-146a_st	miRNA	0.032603	0.591	DOWN
hsa-miR-421_st	miRNA	0.048753	0.623	DOWN
hsa-let-7c_st	miRNA	0.015405	0.674	DOWN
hsa-miR-26a_st	miRNA	0.032863	0.743	DOWN
hsa-let-7d_st	miRNA	0.032690	0.769	DOWN
HBII-85-20_x_st	snoRNA	0.042958	1.300	UP
14qII-1_x_st	snoRNA	0.032863	1.330	UP
ENSG00000200307_st	snoRNA	0.048753	1.355	UP
ACA36_x_st	snoRNA	0.035892	1.419	UP
hsa-miR-93-star_st	miRNA	0.020493	1.425	UP
ENSG00000200377_st	snoRNA	0.001828	1.438	UP
U84_st	snoRNA	0.044495	1.465	UP
hsa-miR-425-star_st	miRNA	0.016454	1.484	UP
hsa-miR-345_st	miRNA	0.032863	1.539	UP
HBII-180C_st	snoRNA	0.048152	1.539	UP
hsa-miR-1228-star_st	miRNA	0.040755	1.542	UP
U53_st	snoRNA	0.032863	1.590	UP
U43_x_st	snoRNA	0.031213	1.591	UP
U43_st	snoRNA	0.031213	1.605	UP
ENSG00000212523_x_st	snoRNA	0.031213	1.618	UP
hsa-miR-423-3p_st	miRNA	0.017498	1.622	UP
HBII-289_st	snoRNA	0.025374	1.646	UP
hsa-miR-339-5p_st	miRNA	0.006466	1.657	UP
U52_st	snoRNA	0.016454	1.657	UP
HBII-180A_x_st	snoRNA	0.039280	1.667	UP
U83_st	snoRNA	0.046181	1.692	UP
hsa-miR-744_st	miRNA	0.017561	1.694	UP
hsa-miR-330-3p_st	miRNA	0.025221	1.736	UP
hsa-miR-1307_st	miRNA	0.015405	1.790	UP
hsa-miR-941_st	miRNA	0.040513	1.880	UP
hsa-miR-944_st	miRNA	0.009982	1.880	UP
hsa-miR-491-5p_st	miRNA	0.020920	1.884	UP
U25_st	snoRNA	0.005814	1.889	UP
hsa-miR-23a-star_st	miRNA	0.028898	1.915	UP
hsa-miR-501-5p_st	miRNA	0.004144	1.951	UP
U91_s_st	snoRNA	0.006699	1.995	UP
hsa-miR-500_st	miRNA	0.001976	2.000	UP
hsa-miR-432_st	miRNA	0.023427	2.030	UP
hsa-miR-1825_st	miRNA	0.015405	2.093	UP
hsa-miR-193b-star_st	miRNA	0.015405	2.386	UP
hsa-miR-1281_st	miRNA	0.004641	2.509	UP
hsa-miR-1228_st	miRNA	0.000146	2.948	UP
hsa-miR-198_st	miRNA	0.000239	5.170	UP
hsa-miR-551b-star_st	miRNA	0.000443	5.398	UP

Then, we wanted to compare whether there was any overlap between the sncRNA identified with correlation analysis and differential expression analysis and we found that 25% of the miRNA and 6% of the snoRNA were common between both analyses (Figure 3).

### In silico analysis

**Figure 4:**
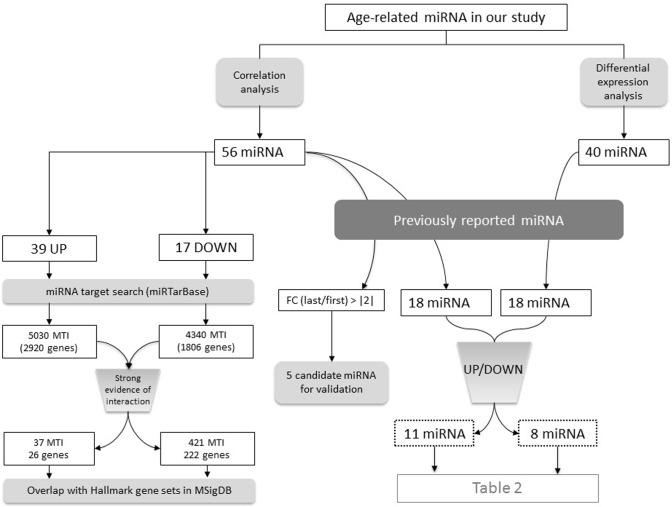
Summary of the number of interesting miRNA identified in each analysis step.

After identifying age-related miRNAs, we wanted to see whether those miRNAs had already been associated to aging. Among the 56 miRNAs that progressively change with age (correlation analysis), 18 had already been associated with aging process and interestingly, 11 had the same expression pattern as in the present study (increased or decreased with age). When we move to the 40 differentially expressed miRNAs between extremes of the cohort (older vs young, differential expression analysis) and compare them with the database, we also found 18 overlapping miRNAs, 8 being in the same direction of deregulation (Figure 4 and Table 3).

**Table 3:** List of miRNA and snoRNA identified in our study which have already been associated to aging according to Digital Aging Atlas database and Serna et al (Serna et al. 2012) and show the same trend with age as in the present paper. Corr: correlation analysis. Diff exp: differential expression analysis.

miRNA/snoRNA	Analysis	DAA Identifier	Change with age	Tissues (DAA or original article)	References
let-7a	SW & Diff exp	DAA2533	decreases	Foreskin	Hackl et al. (2010)
let-7a	SW & Diff exp	NA^1^	decreases	PBMC	Serna et al. (2012)
let-7g	SW & Diff exp	DAA2476	decreases	Foreskin	Hackl et al. (2010)
let-7g	SW & Diff exp	NA^1^	decreases	PBMC	Serna et al. (2012)
let-7i	SW & Diff exp	DAA2561	decreases	T Cell	Hackl et al. (2010)
let-7i	SW & Diff exp	DAA2503	decreases	Foreskin	Hackl et al. (2010)
miR-125b	SW	DAA2517	decreases	Foreskin	Hackl et al. (2010)
miR-126	SW & Diff exp	DAA2605	decreases	Blood	ElSharawy et al. (2012)
miR-126	SW & Diff exp	DAA2504	decreases	Foreskin	Hackl et al. (2010)
miR-1281	SW & Diff exp	NA^1^	increases	PBMC	Serna et al. (2012)
miR-130a	SW	DAA2471	decreases	Foreskin	Hackl et al. (2010)
miR-130a	SW	DAA2006	decreases	Blood, Immune System	Noren Hooten et al. (2010)
miR-15a	SW	DAA2553	decreases	T Cell	Hackl et al. (2010)
miR-19b	SW	DAA2548	decreases	T Cell	Hackl et al. (2010)
miR-19b	SW	NA^1^	decreases	PBMC	Serna et al. (2012)
miR-26a	Diff exp	NA^1^	decreases	PBMC	Serna et al. (2012)
miR-30b	SW	DAA2486	decreases	Foreskin	Hackl et al. (2010)
miR-423-3p	SW & Diff exp	DAA2545	increases	Foreskin	Hackl et al. (2010)
miR-423-3p	SW & Diff exp	NA^1^	increases	PBMC	Serna et al. (2012)
miR-98	Diff exp	DAA2576	decreases	T Cell	Hackl et al. (2010)
U43	SW & Diff exp	NA^1^	increases	PBMC	Serna et al. (2012)
U52	Diff exp	NA^1^	increases	PBMC	Serna et al. (2012)
U91	SW & Diff exp	NA^1^	increases	PBMC	Serna et al. (2012)

^1^The article by Serna et al is not included in Digital Aging Atlas.

As stated before, the main function of miRNAs is to regulate gene expression post-transcriptionally and they act binding to their target mRNAs and causing mRNA degradation or translation inhibition. Therefore, to know which functions were affected by the 56 miRNAs that progressively increase or decrease with age, we searched them in miRTarBase database and found 9370 interactions for 54 miRNA with 4082 genes. Among all those interactions, 458 were supported by strong experimental evidences and included 222 unique genes regulated by miRNAs that decrease with age and 26 unique genes regulated by miRNAs that increase with age (Figure 4). These target genes are involved in immune, cell-cycle and cancer related processes, UV- d image response and other biological processes (Table 4).

**Table 4:** List of gene sets that significantly overlap with target genes of age-related miRNA identified by correlation analysis. The overlap analysis has been made using Hallmark gene sets in Molecular Signature Database v5.1.

Gene Set Name	# Genes in Gene Set (K)	Description	# Genes in Overlap (k)	k/K	p-value	FDR q-value
**TARGETS OF 17 DECREASING miRNA**						
G2M_CHECKPOINT	200	Genes involved in the G2/M checkpoint, as in progression through the cell division cycle.	20	0.1	1.38E-20	6.89E-19
E2F_TARGETS	200	Genes encoding cell cycle related targets of E2F transcription factors.	17	0.085	1.78E-16	2.97E-15
P53_PATHWAY	200	Genes involved in p53 pathways and networks.	17	0.085	1.78E-16	2.97E-15
EPITHELIAL_MESENCHYMAL_TRANSITION	200	Genes defining epithelial-mesenchymal transition, as in wound healing, fibrosis and metastasis.	15	0.075	7.04E-14	5.86E-13
IL2_STAT5_SIGNALING	200	Genes up-regulated by STAT5 in response to IL2 stimulation.	15	0.075	7.04E-14	5.86E-13
TNFA_SIGNALING_VIA_NFKB	200	Genes regulated by NF-kB in response to TNF [GeneID=7124].	15	0.075	7.04E-14	5.86E-13
ALLOGRAFT_REJECTION	200	Genes up-regulated during transplant rejection.	14	0.07	1.25E-12	8.22E-12
APOPTOSIS	161	Genes mediating programmed cell death (apoptosis) by activation of caspases.	13	0.0807	1.31E-12	8.22E-12
UV_RESPONSE_DN	144	Genes down-regulated in response to ultraviolet (UV) radiation.	12	0.0833	6.72E-12	3.73E-11
PI3K_AKT_MTOR_SIGNALING	105	Genes up-regulated by activation of the PI3K/AKT/mTOR pathway.	10	0.0952	1.04E-10	5.22E-10
INFLAMMATORY_RESPONSE	200	Genes defining inflammatory response.	12	0.06	3.13E-10	1.42E-09
KRAS_SIGNALING_UP	200	Genes up-regulated by KRAS activation.	11	0.055	4.34E-09	1.81E-08
APICAL_JUNCTION	200	Genes encoding components of apical junction complex.	10	0.05	5.47E-08	2.10E-07
COAGULATION	138	Genes encoding components of blood coagulation system; also up-regulated in platelets.	8	0.058	3.91E-07	1.40E-06
ADIPOGENESIS	200	Genes up-regulated during adipocyte differentiation (adipogenesis).	9	0.045	6.21E-07	1.72E-06
COMPLEMENT	200	Genes encoding components of the complement system, which is part of the innate immune system.	9	0.045	6.21E-07	1.72E-06
HYPOXIA	200	Genes up-regulated in response to low oxygen levels (hypoxia).	9	0.045	6.21E-07	1.72E-06
INTERFERON_GAMMA_RESPONSE	200	Genes up-regulated in response to IFNG [GeneID=3458].	9	0.045	6.21E-07	1.72E-06
UV_RESPONSE_UP	158	Genes up-regulated in response to ultraviolet (UV) radiation.	8	0.0506	1.09E-06	2.87E-06
IL6_JAK_STAT3_SIGNALING	87	Genes up-regulated by IL6 [GeneID=3569] via STAT3 [GeneID=6774], e.g., during acute phase response.	6	0.069	4.22E-06	1.05E-05
** TARGETS OF 39 INCREASING miRNA**						
TNFA_SIGNALING_VIA_NFKB	200	Genes regulated by NF-kB in response to TNF [GeneID=7124].	6	0.03	1.35E-09	6.75E-08
IL2_STAT5_SIGNALING	200	Genes up-regulated by STAT5 in response to IL2 stimulation.	4	0.02	4.83E-06	1.21E-04
UV_RESPONSE_DN	144	Genes down-regulated in response to ultraviolet (UV) radiation.	3	0.0208	7.43E-05	1.24E-03
APOPTOSIS	161	Genes mediating programmed cell death (apoptosis) by activation of caspases.	3	0.0186	1.03E-04	1.29E-03
EPITHELIAL_MESENCHYMAL_TRANSITION	200	Genes defining epithelial-mesenchymal transition, as in wound healing, fibrosis and metastasis.	3	0.015	1.96E-04	1.40E-03
ESTROGEN_RESPONSE_EARLY	200	Genes defining early response to estrogen.	3	0.015	1.96E-04	1.40E-03
G2M_CHECKPOINT	200	Genes involved in the G2/M checkpoint, as in progression through the cell division cycle.	3	0.015	1.96E-04	1.40E-03
IL6_JAK_STAT3_SIGNALING	87	Genes up-regulated by IL6 [GeneID=3569] via STAT3 [GeneID=6774], e.g., during acute phase response.	2	0.023	1.12E-03	6.99E-03
PI3K_AKT_MTOR_SIGNALING	105	Genes up-regulated by activation of the PI3K/AKT/mTOR pathway.	2	0.019	1.62E-03	9.01E-03
COAGULATION	138	Genes encoding components of blood coagulation system; also up-regulated in platelets.	2	0.0145	2.78E-03	1.39E-02
ALLOGRAFT_REJECTION	200	Genes up-regulated during transplant rejection.	2	0.01	5.72E-03	1.68E-02
E2F_TARGETS	200	Genes encoding cell cycle related targets of E2F transcription factors.	2	0.01	5.72E-03	1.68E-02
GLYCOLYSIS	200	Genes encoding proteins involved in glycolysis and gluconeogenesis.	2	0.01	5.72E-03	1.68E-02
HYPOXIA	200	Genes up-regulated in response to low oxygen levels (hypoxia).	2	0.01	5.72E-03	1.68E-02
INFLAMMATORY_RESPONSE	200	Genes defining inflammatory response.	2	0.01	5.72E-03	1.68E-02
MYOGENESIS	200	Genes involved in development of skeletal muscle (myogenesis).	2	0.01	5.72E-03	1.68E-02
P53_PATHWAY	200	Genes involved in p53 pathways and networks.	2	0.01	5.72E-03	1.68E-02

As for age-related snoRNA, even if they are not included in Digital Aging Database, there are three (U43, U52 and U91) which had already been related to aging process by other authors (Table 3).

### Validation of candidate miRNA

Once we identified a subset of miRNA and snoRNA related to human aging in leucocytes, we validated some candidates in an independent cohort. The selection of the miRNA for validation was made on the basis of fold-change and previous association with aging. There-fore, taking the list of 69 sncRNA that showed to be correlated with aging, we selected those having a fold change higher than 2 (up or downregulated) between the last and the first sliding window, which yielded a list of 15 sncRNA. Finally, we applied an additional filter, selecting those sncRNA that had already been associated to aging according to Digital Aging Atlas. This selection resulted in a list of 5 candidate miRNA: miR-15a, miR-30b, let-7i, let-7g and miR-1281 (Table 5).

**Table 5:** Results for the 5 candidated miRNA selected for validation. Relative quantification (RQ) calculated with RT-qPCR data as well as p-value are shown for both the discovery and validation cohort. For the discovery cohort, microarray results are also shown for comparison purposes.

	discovery cohort							validation cohort	
ProbeSet Name	miRNA	R-SW	last/first (SW)	FC (elder/young) LIMMA	adj.P.Val LIMMA	RQ (Qpcr)	p-value	RQ (Qpcr)	p-value
hsa-miR-1281_st	miR-1281	0.921	2.778	2.509	4.64E-03	1.127	6.60E-01	-	-
hsa-let-7i_st	let-7i	−0.853	0.498	0.455	4.64E-03	0.566	2.86E-02	0.226	1.53E-07
hsa-miR-30b_st	miR-30b	−0.839	0.476	0.503	1.05E-01	0.777	1.07E-01	0.419	7.66E-07
hsa-miR-15a_st	miR-15a	−0.874	0.465	0.525	9.39E-02	0.632	1.21E-01	0.219	1.23E-07
hsa-let-7g_st	let-7g	−0.899	0.434	0.468	1.54E-02	0.578	1.14E-02	0.199	1.05E-08

**Figure 5:**
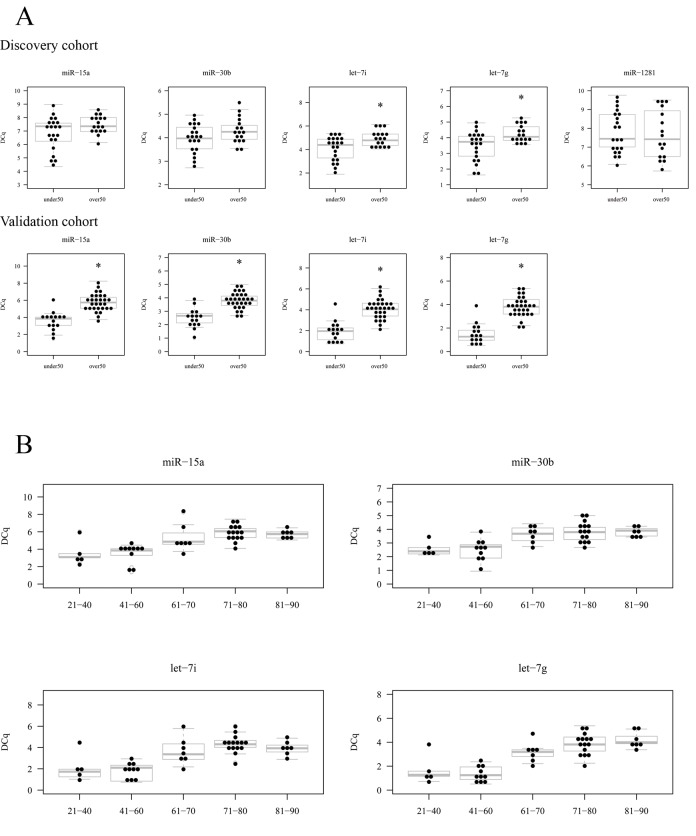
Results of the validation experiment of candidate miRNA. (**A**) The expression of candidate miRNA in elder (over 50) and young (under 50) is shown measured by RT-qPCR in the discovery cohort (upper row) and validation cohort (lower row). In the validation cohort miR-1281 was not analysed. (**B**) The expression of the validated 4 miRNA with age ranges.

First, we assessed by RT-qPCR the expression of candidate miRNA in the same discovery cohort of microarray analysis as a technical validation, which showed that the expression of let-7i and let-7g was significantly lower in the group which was over 50 years (RQ=0.566; p-value= 0.0286 and RQ= 0.578; p-value=0.0281, respectively) (Table 5). miR-15a and miR-30b also had a slightly lower expression in this group, but the difference was not statistically significant (RQ=0.632; p-value=0.121 and RQ=0.777; p-value=0.0591, respectively) (Table 5). On the other hand, miR-1281 did not show any difference in its expression between the two groups (RQ=1.127; p-value=0.672) (Table 5). This RT-qPCR analysis confirmed the results observed in microarray experiment, except for miR-1281 (Figure 5). Even if miR-15a and miR-30b did not show a statistically significant change, we decided to include them in further validation experiments. Therefore, we studied the expression of the four downregulated miRNA in an independent cohort and found a statistically significant underexpression in subjects being older than 50 years (Figure 5A and Table 5).

Furthermore, we segregated the cohort in five age ranges to see whether the expression decrease was a gradual process with age, as identified by microarray experiment. We created 5 groups as follows: 24-40 (n=5), 41-60 (n=10), 61-70 (n=7), 71-80 (n=15) and 81-90 (n=7). Then, we represented the expression values of each miRNA (in DCq) for each group and it can be observed that their expression decreases gradually in this ranges, specially in miR-15a, let-7i and let-7g. However, it is true that the group composed of individuals between 80 and 90 years old, have a similar expression to that of 71-80 (Figure 5B).

## DISCUSSION

In the present work we have identified a sncRNA signature that shows a progressive expression change with age in peripheral blood leucocytes. This signature involves 69 sncRNAs (56 miRNAs and 13 snoRNAs), 48 of them showing an increasing expression while 21 exhibit a decreasing expression pattern. Our data present that the expression of this sncRNAs subset starts to change around 40 years old and at 47 years, this alteration suffers and acceleration, the main change occurring from 47 to 54 years old. These results highlight that the expression change occurring with age is a progressive process rather than being a sudden event happening in the elderly. Yet, it is true that this progressive change does not follow a uniform pattern, given that we have observed an acceleration between 47 to 54 years.

This expression pattern described for sncRNAs, is similar to that found with mRNA expression analysis previously by our group [8]. Both studies have found a subset of RNAs whose expression changes progressively with chronological age. Yet, the most prominent change described for mRNA starts at a later age than that seen in small RNAs (49 years old in transcriptome and 47 in miRNome) and it is slightly more pronounced. Furthermore, both works determine the critical age in the sixth decade of life, based on the age at which increasing and decreasing mRNA and sncRNA cross (Figure 2B).

The correlation analysis carried out in the present work has been able to identify a subset of 69 probes, the expression of which gradually changes with chronological age. Furthermore, a more classical differential expression analysis has stood out an overlap of 35.2% and 25% of miRNA and snoRNA, respectively, with the correlation analysis, highlighting the validity of the correlation analysis to identify age-related miRNA. It is true, however, that there is an important fraction of sncRNA that have been identified only with one of the analyses. This observation suggests that each of the methods is able to address different questions: the correlation analysis would identify genes showing a gradual expression change correlated with chronological age, and would fail to detect those that suffer a big sudden change in the elderly, whereas differential expression analysis would identify genes presenting the opposite situation.

Among the 71 miRNAs and 24 snoRNAs identified in the present study (with both types of analysis), 46 have previously been reported to change with age by different authors and in different tissues or cell types. Interestingly, 16 sncRNA identified in the present study are in the same direction of change as reported previously (Table 3). Surprisingly, some of those miRNAs had been identified in an *in vitro* model of foreskin and our research highlights that those miRNAs may have an impact also in the aging of immune system cells. The rest of the miRNAs had also been described in blood cells, reinforcing the role of those miRNAs in senescence and reflecting the validity of our approach. Of note, let-7a, let-7g, let-7i, miR-126, miR-130a and miR-19b have been reported to be decreased and miR-423-3p increased in the elderly by more than one author apart from the present study, supporting their role in healthy aging (Table 3). This observation, points at those miRNAs as candidate molecules to be studied in the future in order to stablish their biological function and understand their implication in human healthy aging.

Moreover, we have confirmed the decreasing expression of miR-15a, miR-30b, let-7i and let-7g with age in and independent cohort by RT-qPCR. As stated before, those miRNA had already been associated to human aging by several authors. Hackl and colleagues, for instance, had also found the downregulation of these miRNA in several cell types and tissues including T cells, which is in accordance with our results [22]. Serna et al, had also related those miRNA to human aging but, they found and overexpression of miR-30b, miR-15a and let-7i in centenarians compared to octogenarians[17]. These data show the opposite trend than what we have observed in our cohort, where those miRNA appear to decrease with age. Nonetheless, they have studied centenarians, octogenarians and young people and they conclude that as for miRNA expression is concerned, centenarians are more similar to young people than to octogenarians. Therefore, the different trend they observed in miR-30b, miR-15a and let-7i expression comparing to our results could be conferred to the fact that the expression of those miRNA in centenarians is very similar to young people.

In order to assess the possible implication of age-related miRNA in human aging, miRNA-target interaction analysis was carried out, which revealed that age associated miRNAs regulate genes involved in immune, cell-cycle and cancer related processes, UV- induced damage response and others. Interestingly, many of the enriched pathways in age-related decreasing miRNA target genes are the same as those found among increasing miRNA targets. Therefore, those biological processes might be regulated either by repressing the expression (through increasing miRNA) of some genes or reducing miRNA-exerted repression (through decreasing miRNA) of some other genes in the same pathways.

Inflammatory pathways had already been associated to aging process [1,23] and our results indicate that some of those pathways are regulated by miRNAs. For instance, NFKB1 appears to be a target of miR-146a-5p, which decreases with chronological age, being a candidate that would explain the increase in this transcription factor activity that have already been described in immunosenescence[6]. The increase of IL-6 and TNFα plasma levels have been described in elder people as well [24–28], and our results point out that IL-6 and TNFα induced signalling pathways via NFkB might be regulated by a subset of miRNAs.

Moreover, the target genes of miRNAs that exhibit a gradual change with chronological age (found in miRTarBase and before filtering by strong evidence of interaction) include several genes whose expression also show a progressive pattern with age, as found in our previous work [8]. Namely, 40 genes (21 downregulated and 19 upregulated) appear to be age-related miRNA targets and show a concordant progressive expression change (increasing miRNA and decreasing target gene, and vice versa) (Figure 4). This observation points out that age-related miRNAs identified in the present work might be responsible for the expression pattern described for a subset of protein-coding genes.

In conclusion, our study identifies a subset of 69 small non-coding RNAs (54 miRNAs and 15 snoRNAs) of which expression in peripheral blood leucocytes changes progressively with chronological age. Furthermore, our data suggest that the age range from 47 to 54 is critical from a molecular point of view, given that sncRNA expression changes start to accelerate. Moreover, these miRNAs regulate several genes that are involved mainly in cancer and immune system-related pathways, which had already been associated to human aging. Therefore aging could be studied as a result of progressive molecular changes, and different age ranges should be analysed to cover the whole aging process.

## MATERIALS AND METHODS

### Sample donors and sample collection

In order to perform the study, healthy donors were recruited in the Department of Neurology at Donostia University Hospital and the Basque Biobank. The discovery cohort included 38 donors with an age range from 24 to 79 and the validation cohort included 44 donors ranging from 24 to 87 years old. Donors who were over 60 years were examined by a neurologist to ensure their healthy status. Samples from all donors were collected after receiving written informed consent. The study was approved by the hospital's ethics committee.

Peripheral blood (10 ml) was collected by venipuncture after receiving informed consent. Blood samples were collected in tubes containing EDTA as anticoagulant (Vacutainer, Becton Dickinson) and samples were processed and stored at the Basque Biobank.

### RNA isolation

Total RNA was isolated from peripheral blood leucocytes with the LeukoLOCK kit (Ambion, ThermoFisher Scientific, 168 Third Avenue, Waltham, MA 02451, USA) using the alternative protocol to capture small RNAs. RNA concentration was measured using a NanoDrop ND-1000 spectrophotometer and RNA integrity was assessed using Agilent 2100 Bioanalyzer with the RNA 6000 Nano Assay Protocol (Agilent Technologies, 5301 Stevens Creek Blvd. Santa Clara, CA 95051, USA). We only included samples with a RNA integrity number higher than 6.

### sncRNA expression

Total RNA (500 ng) was labeled using the FlashTag Biotin labeling kit (Genisphere LLC, 2801 Sterling Drive, Hatfield, PA 19440, USA) and hybridized to the GeneChip miRNA 1.0 Array (Affymetrix), which covers 847 human miRNAs and 922 human snoRNAs, following manufacturer's instructions. Briefly, RNA molecules were polyadenylated and a biotin-labeled DNA molecule was attached in a subsequent ligation step. Finally, labeled RNA was hybridized to the array, washed and stained in a GeneChip Fluidics Station 450 and scanned in a GeneChip Scanner 7G (Affymetrix, 3420 Central Expressway, Santa Clara, CA 95051, USA).

### Microarray data analysis

#### Data normalization and filtering

We first performed raw data analysis, including a detection step (a probe set is detected above background with an associated p-value) resulting in a true/false call and a quantile normalization step using the miRNA QC Tool software v1.0.33.0 (Affymetrix, 3420 Central Expressway, Santa Clara, CA 95051, USA). In a subsequent filtering step, all non-human probesets were removed, resulting in a final list of 1769 probesets for downstream analysis. The data discussed in this publication have been deposited in NCBI's Gene Expression Omnibus [29] and are accessible through GEO Series accession number GSE89042 (www.ncbi.nlm.nih.gov/geo/query/acc.cgi?acc=GSE89042).

#### Correlation analysis

The workflow followed in this study for data analysis is shown in figure 4. In order to detect the genes whose expression progressively increase or decrease with age, the Pearson's correlation was calculated between the age and each of the sncRNA expression. To do that, we first performed a sliding window analysis. Briefly, we ordered the samples by age from the youngest to the oldest, made a group of first 10 samples (first window) and calculated the average expression for each probeset in this window. Then, we slid the window one sample to the right and calculated the average expression for each probeset in this second window. We carried out this computation to a total of 28 windows, obtaining at the end a matrix of 28 values for each probeset, being each value the average expression of 10 samples. Z-scores were computed for this matrix and these values were used for calculating the correlation with age. Finally, to identify the most significant sncRNAs, we selected those having a R > 0.75 and with a > 50% expression difference between the first and the last sliding window.

#### Differential expression analysis

With the aim of identifying any sncRNA that was differentially expressed between elder subjects and young subjects, these two groups were compared using the “limma” package from Bioconductor [30]. According to the correlation analysis, the cut-off to establish the two groups (young and elder) was set at age 50. Differentially expressed sncRNAs were defined as those passing the false discovery rate (FDR) corrected p-value, set at 0.05 (Figure 4). Venn diagrams were created using Venny 2.1 (http://bioinfogp.cnb.csic.es/tools/venny/index.html).

### Search for microRNA targets and biological function

In order to get the targets of miRNAs, miRTarBase database was used, which gathers experimentally validated miRNA-target interactions (MTI) [31]. First, using a tool available at miRandola web (http://atlas.dmi.unict.it/mirandola/index.php) [32] we converted the miRNA names from miRBase v11 (in which the microarray used is based) to miRbase v20 to match the nomenclature of miRTarBase. Afterwards, we found the miRNA-target interactions registered in miRTarBase for the miRNAs of interest and removed the interactions that have weak evidence according to the database. Afterwards, we computed overlaps of miRNA target genes with Hallmark gene sets from Molecular Signature Database v5.0 on Gene Set Enrichment analysis website [33], considering only the resulting top twenty gene sets from significant list (FDR < 0.05).

Finally, we wanted to evaluate whether the miRNAs identified in the present study had already been related to aging process in other works. To do that, we compared our age-related miRNAs with those compiled in Digital Aging Atlas [34] and the article by Serna et al [17], which is not included in this database. The snoRNA are not included in this database either.

### Validation of candidate miRNA

Validation of candidate miRNA were performed by reverse transcription quantitative PCR (RT-qPCR) using miScript PCR system (Qiagen, QIAGEN Strasse 1 40724 Hilden, Germany). Total RNA (200 ng) was reverse transcribed using miScript II Reverse Transcription kit using the HiSpec buffer and following the manufacturer's protocol in a Veriti thermal cycler (Applied Biosystems, ThermoFisher Scientific, 168 Third Avenue, Waltham, MA 02451, USA). Afterwards, qPCR was carried out with miScript SYBR Green PCR kit, adding 3 ng of cDNA as input, as specified in the protocol. We used the following miScript primer Assays to amplify miRNAs of interest: Hs_let-7g_2, Hs_let-7i_1, Hs_1281_1, Hs_miR-15a_1, Hs_miR-30b_1 and Hs_miR-191_1 as the reference gene. The PCR was carried out in a CFX384 thermal cycler (BioRad, 1000 Alfred Nobel Drive, Hercules, CA 94547, USA).

Raw data were processed in Bio-Rad CFX Manager software and the subsequent analysis to calculate relative expression was carried out in Excel software using 2^−DDCT^ method[35]. Statistical analysis and plots were performed in RStudio software version 1.0.44 running R version 3.3.2. To compare the expression of candidate miRNA between donors who were over 50 years and those who were under 50 years old we applied a Wilcoxon runk sum test.
